# CellCraft: an extensible visual programming application for gene regulatory network inference

**DOI:** 10.1093/bioinformatics/btaf684

**Published:** 2025-12-26

**Authors:** Dongmin Shin, Jeonghwan Henry Kim, Rakbin Sung, Junil Kim, Daewon Lee

**Affiliations:** Department of Applied Art and Technology, College of Art and Technology, Chung-Ang University, Anseong 17546, Republic of Korea; Department of Bioinformatics, Soongsil University, Seoul 06978, Republic of Korea; Department of Applied Art and Technology, College of Art and Technology, Chung-Ang University, Anseong 17546, Republic of Korea; Department of Bioinformatics, Soongsil University, Seoul 06978, Republic of Korea; School of Systems Biomedical Science, Soongsil University, Seoul 06978, Republic of Korea; Department of Applied Art and Technology, College of Art and Technology, Chung-Ang University, Anseong 17546, Republic of Korea; School of Art and Technology, College of Art and Technology, Chung-Ang University, Anseong 17546, Republic of Korea

## Abstract

**Summary:**

Reconstructing gene regulatory networks (GRNs) from single-cell RNA sequencing (scRNA-seq) data is fundamental for understanding cellular dynamics at the molecular level but requires sophisticated workflows. Here, we introduce CellCraft, a web-based application designed to streamline GRN inference. CellCraft integrates multiple GRN reconstruction tools, including TENET, within a unified web application featuring an intuitive graphical user interface. Notably, CellCraft provides a visual programming interface that simplifies the design and execution of complex multistep analyses, thereby enhancing accessibility and facilitating the visualization and interpretation of computational experiments. Furthermore, its modular plugin architecture ensures extensibility, enabling the incorporation of newly developed single-cell analysis algorithms. Consequently, CellCraft provides a user-friendly and extensible application for integrative GRN analysis of scRNA-seq datasets.

**Availability and implementation:**

CellCraft is available on GitHub at https://github.com/cxinsys/cellcraft. The source code has been archived on Zenodo at 10.5281/zenodo.17865848.

## 1 Introduction

Inferring gene regulatory networks is essential for understanding the regulatory interactions underlying various cellular processes and diseases. Recent advancements in high-throughput transcriptomic measurement techniques have facilitated systematic extraction of regulatory relationships from gene expression profiles, enabling the development of advanced GRN inference methods for single-cell datasets (Kim *et al.* 2023). These methods use mathematical and statistical frameworks to reconstruct accurate regulatory networks, elucidating the relationships between genes and their regulators (Pratapa *et al.* 2020, Kim *et al.* 2024). SCENIC uses regression coefficients and information from the transcription factor (TF) binding motif to infer regulatory networks and their target genes based on machine learning techniques (Aibar *et al.* 2017). TENET uses the transfer entropy (TE) of information theory to infer causal relationships from scRNA-seq datasets (Kim *et al.* 2021), and has successfully identified critical regulators across diverse biological contexts (Kim *et al.* 2022).

The rapid expansion of single-cell omics has resulted in a wealth of open-source tools dedicated to the in-depth analysis of GRNs (Kim *et al.* 2023). However, integrating and utilizing these tools within integrative software remains challenging due to several limitations. First, steep learning curves often arise from complex user interfaces presenting an overwhelming number of functionalities. Second, architectural constraints hinder scalability and the integration of new methods. Third, ensuring the reproducibility of multistep computational workflows and managing software dependencies can be inadequate or opaque. Collectively, these issues impede user accessibility and restrict the flexibility required for the analysis of large-scale GRNs.

To facilitate efficient inference and exploration of GRNs, we have developed CellCraft, a web-based visual programming application designed specifically for GRN analysis. CellCraft provides an intuitive graphical user interface (GUI) that simplifies complex bioinformatics workflows and substantially reduces the reliance on manual scripting, enabling biomedical researchers without extensive computational expertise to readily perform detailed GRN analysis. Moreover, the plugin system of CellCraft allows straightforward incorporation of external computational tools, further expanding its applicability to diverse research contexts. The current version of CellCraft provides several preinstalled GRN inference tools through its plugin system, such as GENIE3 (Huynh-Thu *et al.* 2010), GRNBOOST2 (Moerman *et al.* 2019), LEAP (Specht and Li 2017), Scribe (Qiu *et al.* 2020), FastSCODE (Matsumoto *et al.* 2017, Sung *et al.* 2025), and TENET (Kim *et al.* 2022, Sung *et al.* 2024).

## 2 Design and features

### 2.1 System architecture

**Figure 1.**
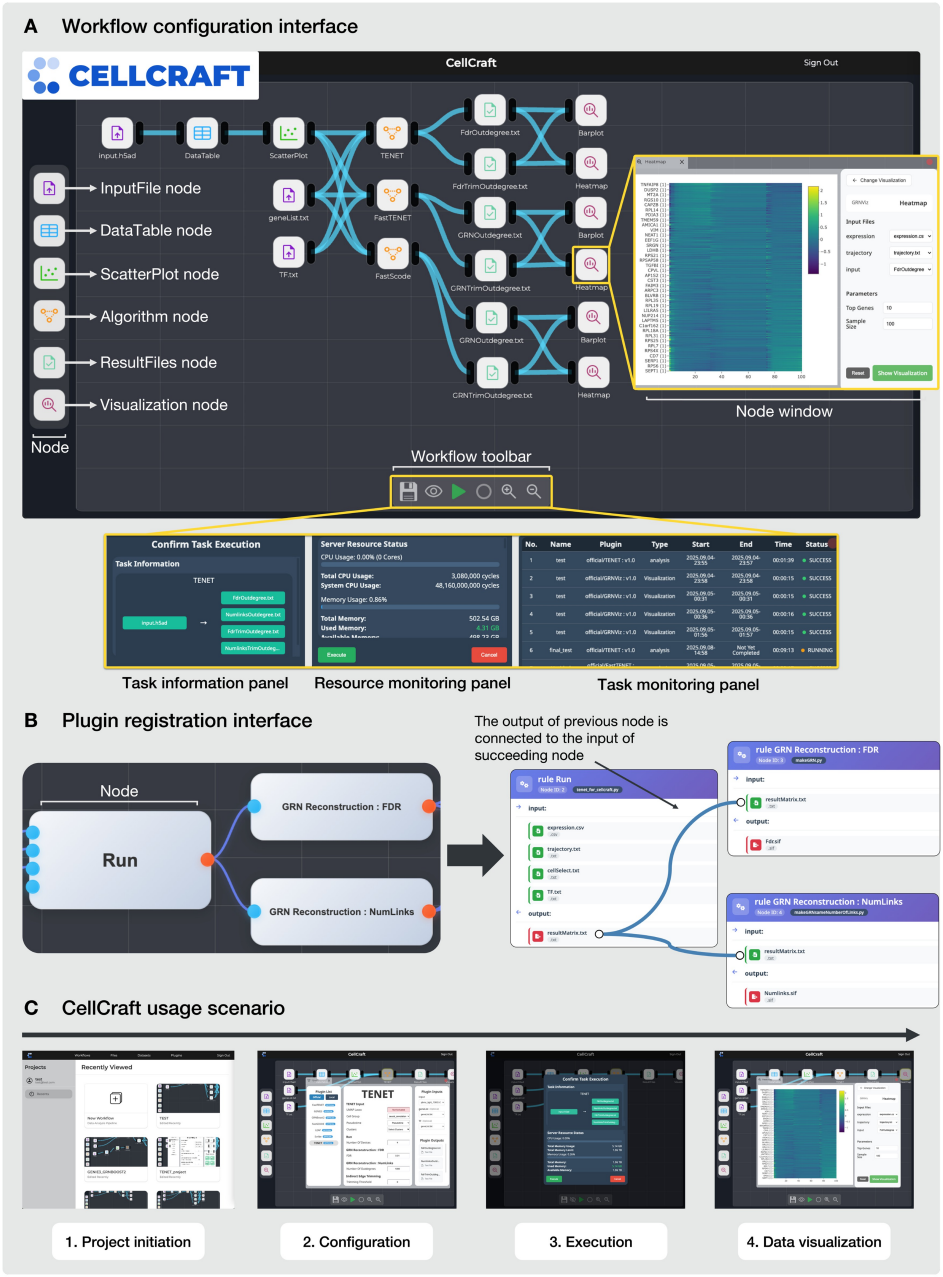
Overview of CellCraft. (A) Interactive visual programming interface for workflow design and execution. (B) Visual interface for registering and managing custom plugins. (C) Representative usage scenario consisting of four steps.

User requests are initiated from a web client built on (You 2014, https://github.com/vuejs/core), which relies on Drawflow (Soler 2020, https://github.com/jerosoler/Drawflow) for visual programming and Plotly (Plotly Technologies Inc. 2015) for data visualization (Fig. 1, available as supplementary data at *Bioinformatics* online). The user requests are routed through an NGINX (Reese 2008) web server to a backend API server implemented with FastAPI (Ramírez 2018, https://github.com/fastapi/fastapi). The FastAPI-based backend serves as the central component of the system, managing user authentication, workflow validation, task execution, and data processing. It stores and retrieves essential information such as user accounts, plugin metadata, workflow configurations, and results from a PostgreSQL (PostgreSQL Global Development Group 1996, https://www.postgresql.org) database via SQLAlchemy (Bayer 2012).

CellCraft uses a distributed task queue system (Figs 1 and 2, available as supplementary data at *Bioinformatics* online). When a user initiates workflow execution, the backend API compiles the user-defined workflow and associated parameters into an executable Snakefile (Mölder *et al.* 2021). This Snakefile is then dispatched as a job via RabbitMQ (RabbitMQ Team 2007, https://www.rabbitmq.com) to a Celery (Solem^ 2009^, https://github.com/celery/celery) task processing server. Celery workers execute the workflow via Snakemake, which coordinates the execution of individual tasks according to the workflow’s directed acyclic graph (DAG). Throughout the execution process, task status and output files are continually stored in the database. Once workflow execution is complete, the results are accessible through the web client via API requests to the backend. The entire system is containerized with Docker (Merkel 2014) to ensure consistent deployment and reproducibility across diverse computing environments.

### 2.2 Visual programming

Visual programming has been adopted in bioinformatics as an approach to manage analytical complexity and improve the accessibility through intuitive user interfaces. Taverna enables researchers to visually compose workflows by integrating local applications or remote web services, emphasizing reproducibility and facilitating the sharing of scientific protocols (Oinn *et al.* 2004). KNIME is an open-source visual analytics platform that enables users to build data analysis pipelines by assembling nodes for data integration, processing, machine learning, and visualization, facilitating data mining tasks without extensive programming expertise (Berthold *et al.* 2007). Galaxy is an actively maintained web-based platform providing an intuitive visual interface for executing diverse bioinformatics tools, highlighting reproducible and collaborative research (Abueg *et al.* 2024).

Compared to general-purpose platforms such as Taverna, KNIME, and Galaxy, CellCraft provides a lightweight visual programming environment dedicated to GRN inference (Fig. 1A). By focusing on essential GRN-related functions, CellCraft maintains a simple and user-friendly design, lowering the learning curve and installation overhead. Consequently, researchers can quickly adopt CellCraft without encountering usability barriers, enabling them to effectively focus on biological insights rather than technical complexities. The visual programming capabilities of CellCraft are implemented by integrating the Drawflow flowchart library for the GUI with the Snakemake workflow management system. In other words, the integration of the Drawflow frontend with the Snakemake backend explicitly defines task dependencies and utilizes the parallel execution capabilities inherent in the DAG structure of the workflow. In the workflow editor, users can interact with nodes that allow direct inspection of data frames (tables), visualization of analysis results, on-the-fly parameter adjustments, and management of input and output files within the visual workflow (Fig. 1A). A key advantage of CellCraft is its visual plugin registration process. Users define inputs, outputs, and parameters for each plugin based on predefined rules. Once registered, each plugin can be incorporated into customizable workflows as a node that represents an executable analysis unit.

### 2.3 Plugin system

The plugin system of CellCraft is designed to provide flexibility and accessibility for researchers aiming to integrate their own computational methods. In typical plugin architectures, users are required to implement plugins using a specific programming language closely tied to the system’s underlying architecture. For example, in Java-dependent software, plugins must be implemented in Java, requiring users unfamiliar with the language to first learn it before adding new plugins (Shannon *et al.* 2003, Berthold *et al.* 2007). In contrast, the CellCraft plugin system simplifies this process substantially by allowing users to register plugins through clearly defined rules specifying inputs, outputs, execution commands, and parameters via a visual plugin registration interface (Fig. 1B and Notes 2.3, available as supplementary data at *Bioinformatics* online). This visual approach provides clear guidance and immediate feedback, facilitating rapid plugin development and reliable deployment, thus helping researchers concentrate primarily on analysis tasks.

### 2.4 Usage scenario

A common usage scenario of CellCraft for GRN inference consists of four steps: (i) Project initiation, (ii) Configuration, (iii) Execution, and (iv) Data visualization (Fig. 1C, Figs N1–N4, available as supplementary data at *Bioinformatics* online).

At the project initiation stage, users upload their own data files via the “Files” page. Users can also utilize the preuploaded datasets in the “Datasets” section. Upon creating a new workflow project, users can either select a template corresponding to a specific GRN inference method or start with an empty project. The template workflow comprises preconfigured nodes with default parameters, serving as a readily executable example that can be further customized as required.

During the configuration stage, users specify input data settings and execution parameters for each node in the workflow. Input data can be explored using “DataTable” and “ScatterPlot” nodes. For instance, users can interactively select subsets of cells using the lasso tool within a 2D UMAP plot. The workflow can then be further expanded by incorporating additional algorithm and visualization nodes.

After initiating the task via the “Run” button, users can monitor task progress in real-time through the “Task” monitoring panel, which displays states such as running, success, failed, or revoked. The execution process of a task can be examined in detail by monitoring the execution status and stepwise logs. Once the task is completed successfully, the data analysis and visualization stage begins. The GRN inference results are automatically loaded into corresponding “ResultFiles” nodes, and users can select the result file they want to analyze. This result file can be connected to visualization nodes and used as an input when generating plots. CellCraft provides built-in visualization options through the “GRNViz” plugin, including interactive bar plots ranking key regulators by out-degree, pseudo-time heatmaps illustrating gene expression dynamics, and network graphs depicting the inferred GRN structure. All result and configuration files can be downloaded for subsequent in-depth analysis using external computational tools.

## 3 Conclusion

CellCraft is a web-based application specifically designed to simplify GRN inference. By integrating various tools through a lightweight visual programming environment and a modular plugin system with a visual registration interface, CellCraft makes sophisticated GRN analysis accessible to researchers regardless of their computational expertise. These features significantly reduce complexity, installation barriers, and the need for extensive programming skills, while empowering users to easily integrate, customize, and deploy analysis workflows suited to their research objectives.

## Supplementary Material

btaf684_Supplementary_Data

## Data Availability

CellCraft is openly accessible at https://cellcraft.app. The source code underlying this article is available on GitHub at https://github.com/cxinsys/cellcraft and archived in Zenodo at https://doi.org/10.5281/zenodo.17865848.
