# Supplementary material for: CellCraft: an extensible visual programming application for gene regulatory network inference
Source: Bioinformatics. 2025 Dec 26;42(1):btaf684. doi: 10.1093/bioinformatics/btaf684 (PMC12858299; doi:10.1093/bioinformatics/btaf684)
Supplement: btaf684_Supplementary_Data [file btaf684_supplementary_data.pdf]

Supplementary Data

CellCraft: an extensible visual programming application for gene regulatory network inference

Dongmin Shin[1], Jeonghwan Henry Kim[2], Rakbin Sung[1],

Junil Kim[2,3], and Daewon Lee[1,4]

[1]Department of Applied Art and Technology, Chung-Ang University, Anseong 17546, Republic of Korea
[2]Department of Bioinformatics, Soongsil University, Seoul 06978, Republic of Korea
[3]School of Systems Biomedical Science, Soongsil University, Seoul 06978, Republic of Korea
[4]School of Art and Technology, Chung-Ang University, Anseong 17546, Republic of Korea

Supplementary Figures

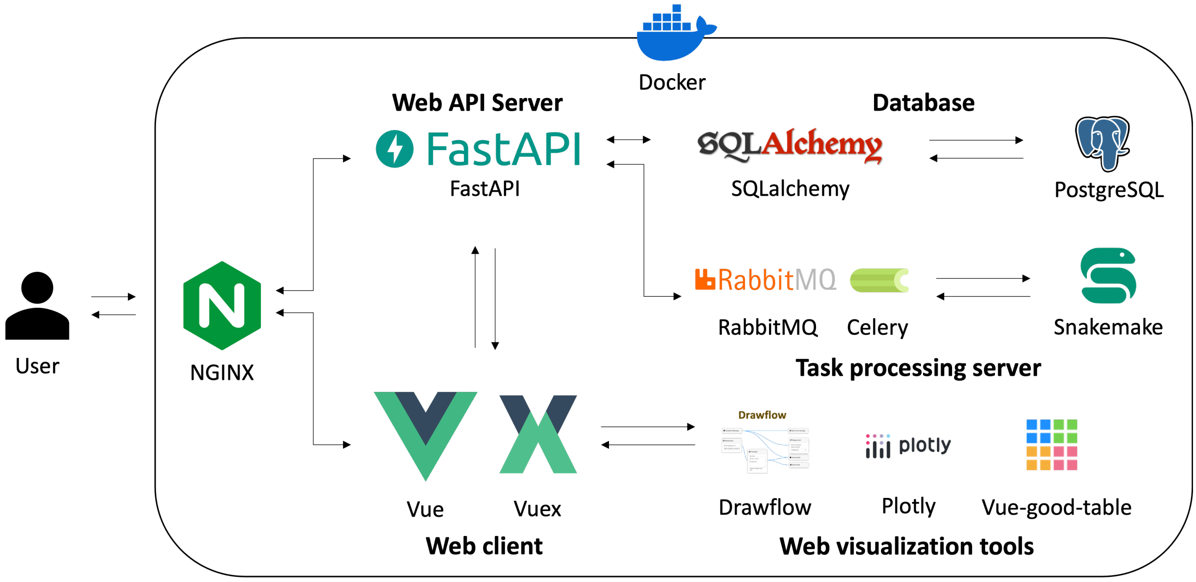

Figure S1. Application architecture of CellCraft. Key components include FastAPI for the backend, SQLAlchemy and PostgreSQL for database management, and RabbitMQ with Celery for task queuing and asynchronous processing. The frontend is developed using Vue.js, with Drawflow for the visual programming interface and Plotly for interactive data visualization. NGINX is used as a web server, and Docker is employed to containerize microservices and plugins.

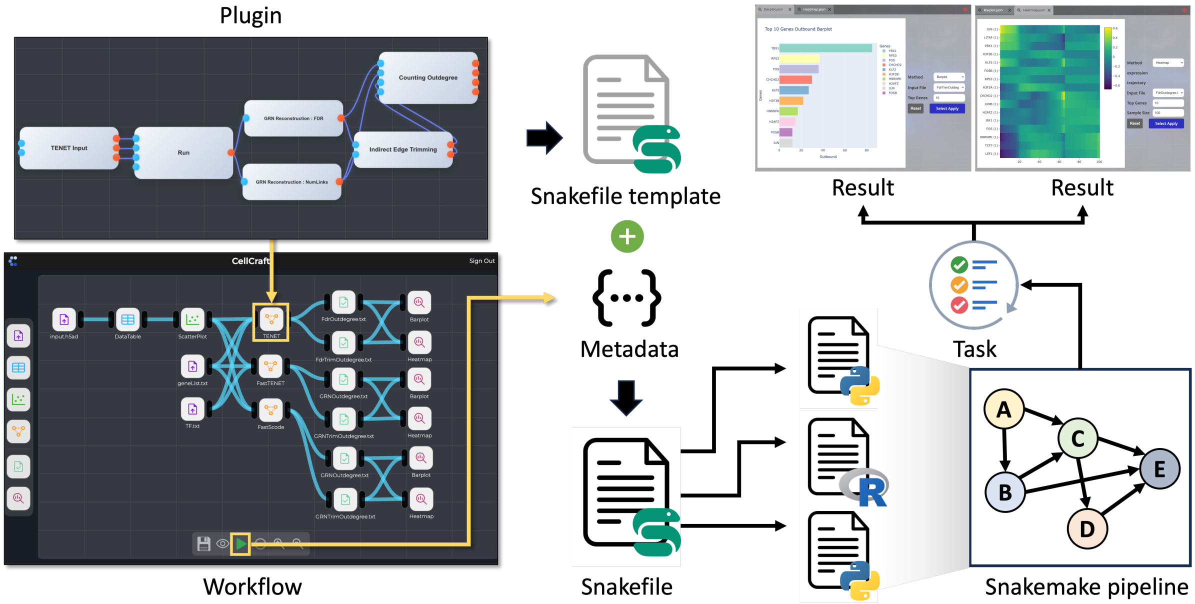

Figure S2. Workflow execution process in CellCraft. CellCraft executes a workflow using a Snakefile template derived from predefined plugins. The parameter configurations specified in the workflow are stored as metadata, which is then utilized to generate the final Snakefile required for execution. The Snakemake pipeline operates as a single task, executing multiple scripts in a directed acyclic graph (DAG) structure based on the Snakefile to generate the target results. Upon task completion, results can be accessed and analyzed through the web interface.

Supplementary Notes

1 Introduction

CellCraft is an open-source project available on its GitHub repository for development and use. The repository includes installation instructions, technical documents, and tutorial videos for quick exploration of the application.

- **GitHub Repositories**:
  - Main project: github.com/cxinsys/cellcraft
  - Official plugins: github.com/cxinsys/cellcraft-plugin
  - Plugin templates: github.com/cxinsys/cellcraft-plugin-templates
- **Tutorial Videos**: youtube.com/@CellCraft-cislab

In these supplementary notes, we present a detailed overview of CellCraft's core functionalities and explain the graphical user interface (GUI) through step-by-step tutorials.

2 Tutorial

2.1 Installing the application

Step 1: Checking system requirements

To install CellCraft locally, users must first verify system requirements. Since CellCraft's analysis tools fundamentally utilize CPU and RAM resources for execution, running the application on hardware that meets the specified requirements is essential. The web application and plugins require approximately 60GB of storage space, and users are advised to ensure at least 100GB of available disk space to accommodate uploaded files and generated outputs during application usage. As CellCraft operates on a Docker-based architecture, it is crucial to install Docker and Docker Compose with versions that match the specified requirements. For users intending to utilize GPU-enabled plugins, it is strongly recommended to configure the environment with compatible NVIDIA Driver and CUDA versions according to the requirements.

Table 1. System Requirements

**Component**	**Minimum**	**Recommended**
CPU	4 cores	8+ cores
RAM	8 GB	16+ GB
Storage	70 GB	100+ GB
OS	Ubuntu 20.04 LTS, Windows 10/11, macOS 10.15 Catalina	Ubuntu 22.04 LTS
OS Kernel	6.8.0	6.8.0+
glibc	2.39	2.39+
Docker	20.10.0	24.0.0+
Docker Compose	v2.0.0	v2.20.0+
NVIDIA Driver	535.171.04	535.171.04+
CUDA	12.1	12.2+

Step 2: Installing with Docker

CellCraft's web application is designed with a microservices architecture, comprising five core components: Web Client (Vue.js), Web API Server (FastAPI), Task Processing Server (Celery), Database (PostgreSQL), and Message Broker (RabbitMQ). Additionally, eight official plugins are managed as

individual Docker containers. Notably, two GPU-enabled plugins (FastTENET and FastSCODE) are configured as optional components to accommodate users without GPU hardware.

The entire system is built and deployed using Docker Compose, with all Docker containers configured as dedicated images and managed through the GitHub Container Registry (GHCR). Previously, users had to build all plugin images locally, which required 2-3 hours due to dependency resolution from multiple upstream sources (Debian, Ubuntu, CRAN, Bioconductor, PyPI). By transitioning all plugins to pre-built GHCR images, we achieved a 95% reduction in plugin setup time to approximately 10-14 minutes.

CellCraft also provides options for installing core services (Frontend, Backend, and Celery workers). Users can choose between local image building or GHCR-based pulling depending on their needs. Table 2 compares setup times across deployment mode (CPU-only vs. GPU-enabled) and build method (Local Build vs. GHCR Pull). For users prioritizing stability and quick deployment, GHCR-based installation is recommended, completing in 10-16 minutes compared to 13-19 minutes for local builds.

The installation process is as follows:

- **Clone the repository:**

  ```
  git clone --recurse-submodules https://github.com/cxinsys/cellcraft.git
  ```

- **Configure plugin submodule (if needed):**
  Check the current submodule status:

  ```
  cd cellcraft/backend/plugin/official && git status
  ```

  Switch to the appropriate branch based on your installation mode:

  ```
  git switch release/plugins-v1.1      # For GPU-enabled installation
  git switch release/plugins-v1.0-cpu  # For CPU-only installation
  ```

- **Start the application:**
  For GPU-enabled installation:

  ```
  cd cellcraft && ./run-gpu-mode.sh
  ```

  For CPU-only installation:

  ```
  cd cellcraft && ./run-cpu-mode.sh
  ```

  If the scripts fail to execute, use these manual commands:
  For GPU-enabled setup (AMD64 with NVIDIA GPU):

  ```
  cd backend/plugin/official && git switch release/plugins-v1.1
  cd ../../.. && docker compose -f docker-compose.gpu.amd64.yml up -d --build
  ```

  For CPU-only setup (AMD64):

  ```
  cd backend/plugin/official && git switch release/plugins-v1.0-cpu
  cd ../../.. && docker compose -f docker-compose.cpu.amd64.yml up -d --build
  ```

  For CPU-only setup (ARM64):

  ```
  cd backend/plugin/official && git switch release/plugins-v1.0-cpu
  cd ../../.. && docker compose -f docker-compose.cpu.arm64.yml up -d --build
  ```

- **Access the application at http://localhost:8080**

Table 2. Comparison of setup times between local build and GHCR deployment.

Configuration	Core Services Build/Pull	Plugin Initialization	Total Time
CPU (Local Build)	3-5 min	9-11 min	13-15 min
GPU (Local Build)	4-6 min	12-14 min	17-19 min
CPU (GHCR Pull)	1-3 min	9-11 min	10-12 min
GPU (GHCR Pull)	1-3 min	12-14 min	14-16 min

Step 3: Verifying the installation

Even after successful installation of the web application via Docker Compose, various issues may arise, such as inaccessibility to the web interface or malfunctioning features. CellCraft provides a diagnostic script to troubleshoot such cases. This script performs Docker container status checks, frontend accessibility tests, backend API connection verification, and plugin image verification. The script first prompts users to select their installation mode (CPU or GPU), then validates all components accordingly. When all checks pass, users receive a clear confirmation message with access URLs. If issues are detected, specific error messages are displayed to identify the cause, along with links to official troubleshooting documentation.

The script executes as follows:

```
$ ./check-installation.sh

================================================
        CellCraft Installation Check
================================================

Select installation mode:
  1) CPU mode (6 plugins)
  2) GPU mode (8 plugins)

Enter choice [1-2]: 2
[INFO] Checking GPU mode installation

[INFO] Checking container status...
[PASS] frontend container running
[PASS] backend container running
[PASS] db container running
[PASS] rabbitmq container running
[PASS] celery container running

[INFO] Testing HTTP endpoints...
[PASS] Frontend accessible (port 8080)
[PASS] Backend API accessible (port 8000)

[INFO] Verifying plugins for GPU mode...
[PASS] All 8 plugin images found
```

2.2 Running GRN inference

This section outlines the steps to perform GRN inference using the available tools within CellCraft. These instructions guide users through the process of configuring and executing a workflow project.

Step 1: Verifying plugin availability

To begin, navigate to the 'Plugins' page and ensure that the required GRN inference tool is installed and activated. For official plugins, the available plugin list varies depending on which deployment mode (CPU-only or GPU-enabled) the user has selected, which should be verified in advance. For local plugins,

if you have completed the plugin addition process, toggle the activation switch to enable it. Ensuring plugin availability is essential for utilizing plugins within a workflow project.

Step 2: Initializing a workflow project

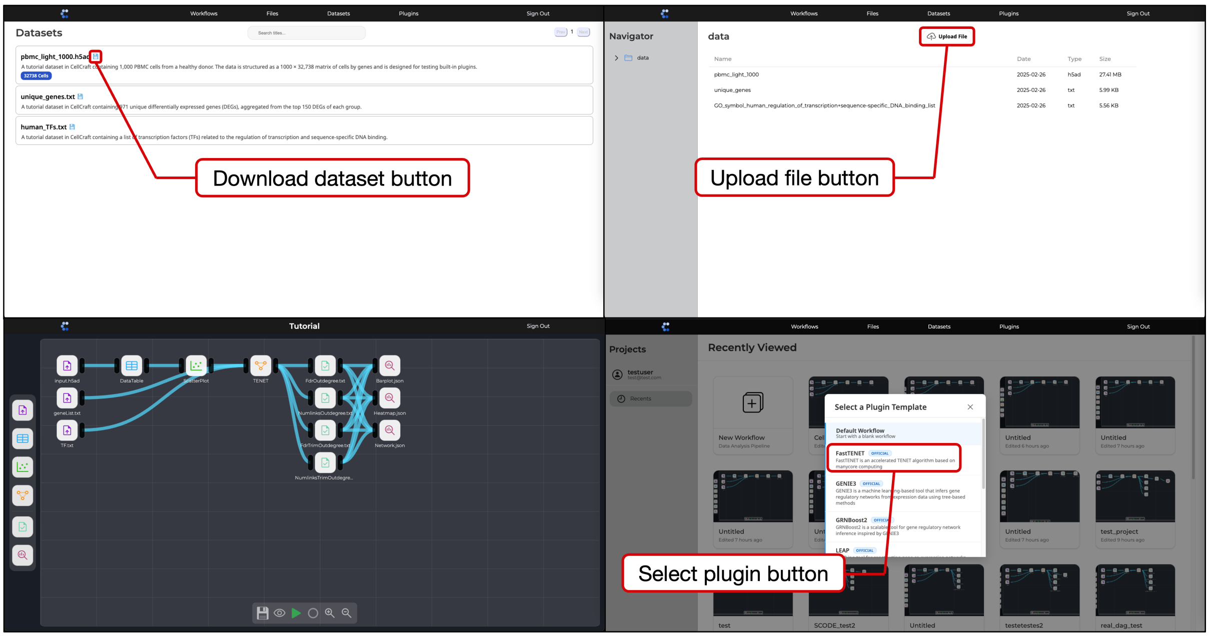

Figure N1. An example of GUIs used for workflow project initialization.

To begin using the GRN inference tool in CellCraft, users must first initialize a workflow project (Figure N1).

The process starts with downloading the tutorial dataset, which is provided to facilitate easy experimentation. This dataset includes:

- **PBMCLight1000.h5ad**: A tutorial dataset containing 1,000 PBMC cells from a healthy donor. The data is structured as a 1,000 × 32,738 matrix of cells by genes and is designed for testing built-in plugins.
- **UniqueGenes.txt**: A tutorial dataset containing 971 unique differentially expressed genes (DEGs), aggregated from the top 150 DEGs of each group.
- **HumanTFs.txt**: A tutorial dataset containing a list of transcription factors (TFs) related to the regulation of transcription and sequence-specific DNA binding.

Once the dataset is downloaded, the next step is to upload the files to CellCraft. Users can do this by navigating to the 'Files' page and uploading the H5AD, CSV, and TXT files individually. Once uploaded, these files will be available for use within a workflow project and can also be managed or deleted as needed.

If no workflow project exists, users can create a new one by clicking the 'New Workflow' button. During this process, they can select an activation plugin template, which automatically initializes the workflow project with the components required for GRN inference.

Step 3: Configuring the workflow project

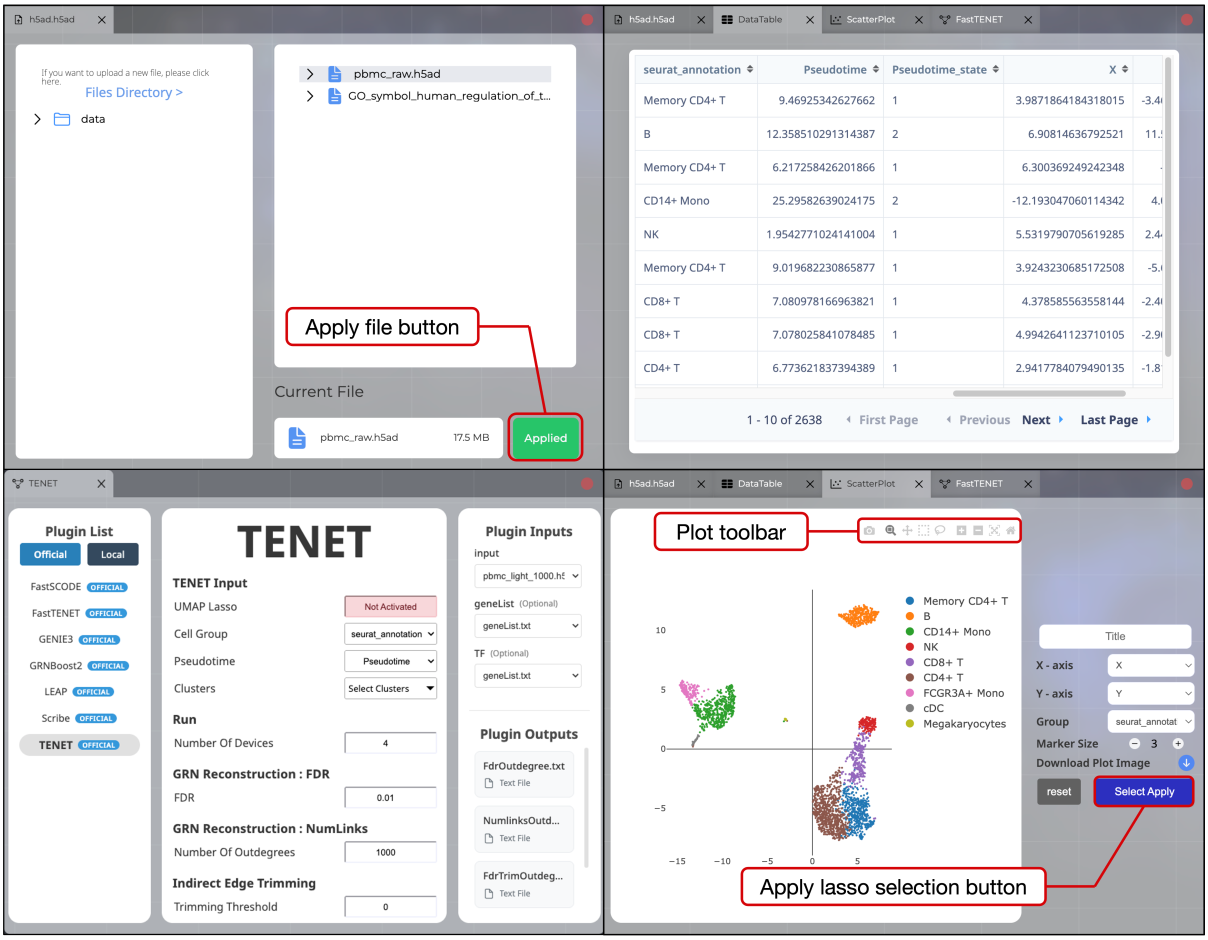

Figure N2. An example of GUIs used for configuring the node window.

After initializing, users must configure the workflow project to prepare it for execution. This involves setting up nodes, assigning input files, exploring and filtering data, and adjusting algorithm parameters (Figure N2).

First, the workflow project is automatically populated with the necessary nodes required for execution. Users can customize their workflow project by renaming the title and saving it for future use.

Next, users must configure the 'InputFile' nodes by assigning the previously uploaded files. These files serve as the foundation for using the functionality of each node.

Once the 'InputFile' nodes are set, users can proceed to explore the configured data using 'DataTable' and 'ScatterPlot' nodes. The 'DataTable' node allows users to explore matrix-based data extracted from the H5AD and CSV files, while the 'ScatterPlot' node provides a graphical representation of gene expression data. Additionally, users can interact with the scatter plot using the 'Lasso Select' tool to define specific regions for analysis.

Finally, users must configure the 'Algorithm' node, which enables tuning of execution settings. Depending on the selected plugin, the parameters are pre-configured with recommended values by default to ensure optimal performance. However, users have the flexibility to modify these parameters based on their specific analysis requirements. If there are connected 'InputFile' nodes, users can select files via dropdown menus to prepare the necessary inputs for the plugin. Additionally, the expected primary output files from the plugin can also be reviewed.

Step 4: Executing the task

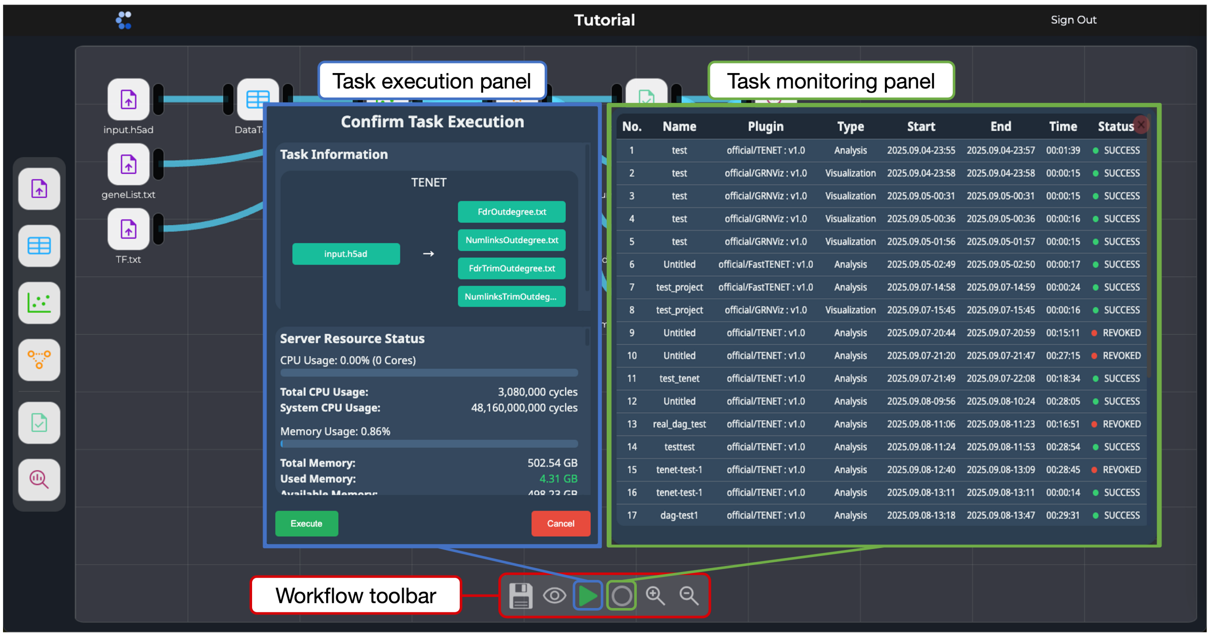

Figure N3. An example of GUIs used for executing the task.

Once the workflow project configuration is complete, users can proceed with executing the task. This process involves initiating task execution and monitoring its progress to ensure successful completion (Figure N3).

To begin, users must click the ‘Run’ button in the ‘Workflow toolbar’. Before execution, a summary window appears showing key task details. This includes the selected input files, expected output files, and system-resource monitoring, providing an overview of the task’s execution settings. Reviewing this information ensures that all configurations are correctly set before proceeding.

After confirming the task details, clicking the ‘Execute’ button will start the task based on the configured workflow project. During execution, users can monitor real-time progress by accessing the ‘Task monitoring’ panel. This panel displays detailed information including the plugin name and version, task type, workflow title, and timestamp, while providing live updates on the task’s execution status through stages such as ‘RUNNING’, ‘SUCCESS’, or ‘FAILURE’. Additionally, the ‘View Logs’ feature allows users to review output logs from the execution script and download them in JSON/TXT format, while the ‘View Progress’ feature enables visual tracking of progress at each pipeline stage. Through these features, users can effectively manage tasks and perform detailed debugging for troubleshooting as needed.

Step 5: Visualizing the result data

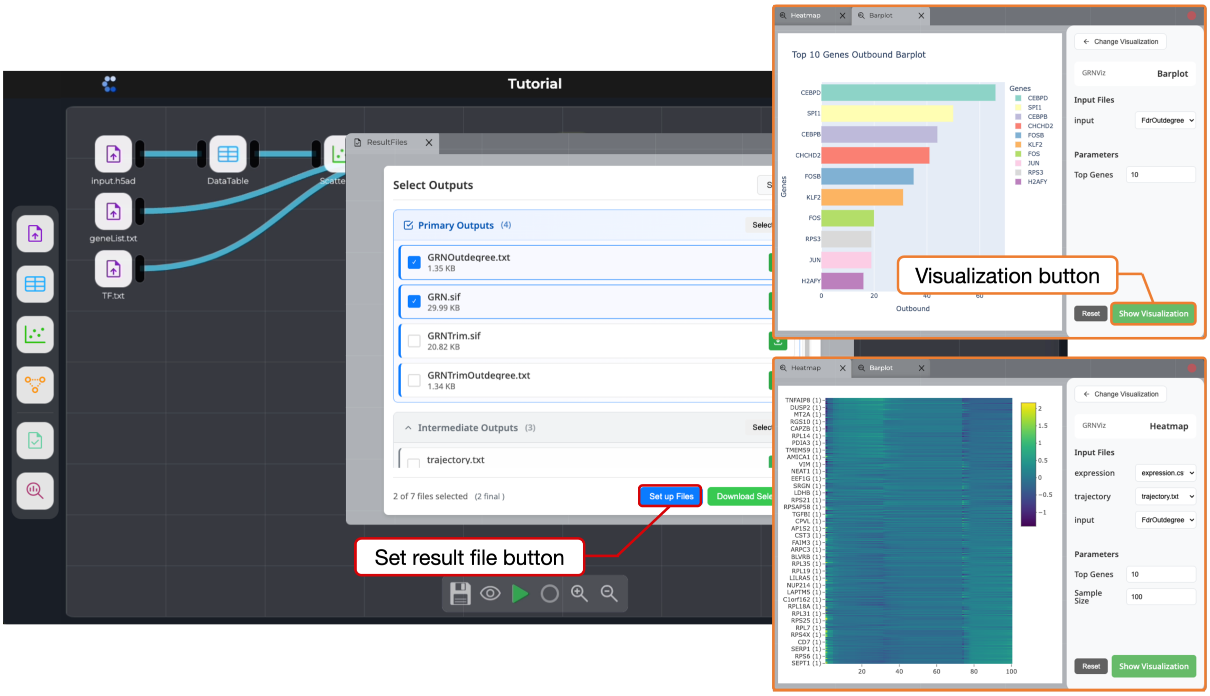

Figure N4. An example of GUIs used for visualizing the result data.

Once the task execution is complete, users can examine the generated output files and visualize key findings (Figure N4).

By checking the 'Task monitoring' panel, users can confirm that the executed task has reached 'SUCCESS' status. At this point, the 'Download Manifest' feature enables users to download the Execution Manifest for successful tasks. The Execution Manifest is a document that records the complete analysis process—including which data (inputs) were used, which plugin version and parameters were applied, and what results (outputs) were generated—thereby ensuring reproducibility of the task.

Users can access output files by connecting 'ResultFiles' nodes to the completed 'Algorithm' node. Output files are categorized and displayed as 'Primary Outputs' and 'Intermediate Outputs', allowing users to select multiple files simultaneously. For visualization purposes, output files must be assigned to 'ResultFiles' nodes and can be downloaded for examination if needed.

CellCraft provides visualization tools through an official plugin called 'GRNViz', which enables users to analyze output data. 'GRNViz' offers the following visualization tools:

- **Bar plot**: Displays gene regulatory influence and ranks genes according to the number of outgoing connections.
- **Heatmap**: Displays gene expression dynamics across pseudotime, highlighting activation and repression patterns.
- **Network**: Displays gene regulatory interactions as a directed network, emphasizing key regulators and their connections.

Users can select their desired visualization tool from the initial GUI screen of the 'Visualization' node. To generate visualizations, users must select an input from the files available in the 'ResultFiles' node connected to the 'Visualization' node and configure the necessary parameters. Once all values are set, users can click the 'Execute Visualization' button to visualize the results. The visualization results are provided interactively, allowing for user interaction, and the resulting images can be downloaded as needed.

2.3 Adding custom plugins

This section outlines the step-by-step process of creating and uploading custom plugins in CellCraft. Custom plugins are classified and managed as Local plugins, distinct from the official plugins supported by

CellCraft. Local plugins are available only in the local installation environment and can be continuously managed through the features provided on the 'Plugins' page.

Step 1: Writing scripts and preparing dependencies

A plugin consists of multiple scripts that are executed sequentially through the Snakemake library. Additionally, the script execution environment is provided through Docker containers built with dependency files. Executing scripts in this manner provides the following advantages:

- **Task independence**: The execution environment configured with Docker containers ensures independence and consistently provides a uniform environment.
- **Reproducibility**: The Snakemake pipeline defined through DAG structure ensures consistent results through clear execution order and identical input configurations.
- **Debugging**: Intermediate outputs help identify and resolve errors efficiently.

```python
import pandas as pd
import sys

exp_matrix_path = sys.argv[1]  # Path to input expression matrix (CSV)
top_n = int(sys.argv[2])       # Number of top genes to select
output_path = sys.argv[3]      # Path to save output file
selected_genes_path = None if sys.argv[4] == 'None' else sys.argv[4]  # Optional: Path to selected genes file

# Load file
df = pd.read_csv(exp_matrix_path, index_col=0)

if selected_genes_path:
    selected_genes = pd.read_csv(selected_genes_path, header=None).squeeze().tolist()
    df = df[selected_genes]

top_genes = df.mean(axis=0).nlargest(top_n).reset_index()
top_genes.columns = ['Gene', 'AverageExpression']

# Save result
top_genes.to_csv(output_path, index=False)
print(f'Top {top_n} genes saved to {output_path}')
```

Listing 1. An example of Python script following CellCraft parameter conventions

```r
args <- commandArgs(trailingOnly = TRUE)

exp_matrix_path <- args[1]  # Path to input expression matrix (CSV)
top_n <- as.integer(args[2])  # Number of top genes to select
output_path <- args[3]  # Path to save output file
selected_genes_path <- ifelse(args[4] == 'None', NA, args[4])  # Optional: Path to selected genes file

# Load file
df <- read.csv(exp_matrix_path, row.names = 1)

if (!is.na(selected_genes_path)) {
    selected_genes <- read.csv(selected_genes_path, header = FALSE)[,1]
    df <- df[, colnames(df) %in% selected_genes]
}

avg_expression <- colMeans(df, na.rm = TRUE)
top_genes <- head(sort(avg_expression, decreasing = TRUE), top_n)
top_genes_df <- data.frame(Gene = names(top_genes), AverageExpression = top_genes)

# Save result
write.csv(top_genes_df, file = output_path, row.names = FALSE)
cat(paste('Top', top_n, 'genes saved to', output_path, "\n"))
```

Listing 2. An example of R script following CellCraft parameter conventions

Scripts must follow parameter conventions to ensure compatibility (Listing 1, Listing 2):

- In **Python**, use `sys.argv[N]` to retrieve arguments.
- In **R**, use `args <- commandArgs(trailingOnly = TRUE)` and `args[N]`.
- Convert non-string data types explicitly (e.g., `int()`, `float()`, `bool()`).
- Optional parameters may receive the string `"None"` when undefined. These must be conditionally handled.

To ensure successful execution, all dependencies must be installed. The standard way to manage dependencies is to list them in a recognized format. For Python, dependency information should be recorded in `requirements.txt` or `environment.yml`, and for R, the `renv.lock` file is recommended. These files allow CellCraft to automatically install the necessary packages from public repositories such as PyPI or CRAN during plugin registration.

In some cases, custom modules that are not available on public repositories may be used. These can be organized as a folder containing source code and uploaded along with the plugin. For example, the following code snippet can be used to append such a local directory to the system path:

```
# Python
current_dir = os.path.dirname(os.path.abspath(__file__))
sys.path.append(current_dir)

from my_libs.my_module import my_func

my_func()

# R
source("my_libs/my_module.R")
my_func()
```

Additionally, for packages that are not available on public package managers and are instead distributed as binary archives (e.g., `.whl`, `.tar.gz`), users may upload these files directly. Such packages will be installed locally within the plugin execution environment. To enable this, the corresponding installation paths must be specified in `requirements.txt`, `environment.yml`, or `renv.lock` assuming the archive files are located in the same directory as these dependency files.

Step 2: Registering the plugin

The plugin registration process involves several key steps (Figure N5 and Figure N6):

1. Specify the plugin name, description, and type.
2. Upload the necessary dependency files.
3. Define nodes using the plugin editor.
4. Review and upload the complete plugin registration.

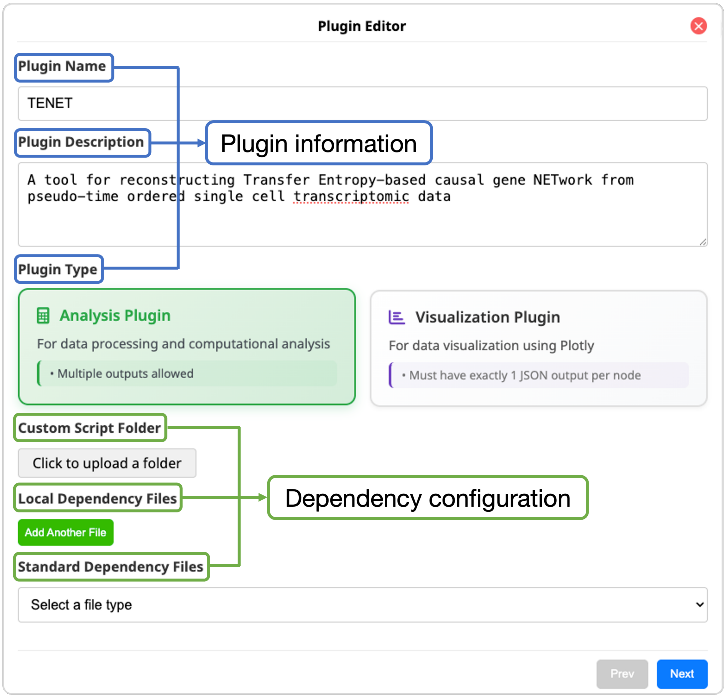

Figure N5. An example of the GUI for plugin registration step 1.

The plugin name and description are essential information that must be provided for users to identify and understand the plugin. Additionally, the plugin type must be selected between 'Analysis' and 'Visualization'. 'Analysis' plugins are used as analytical tools in the 'Algorithm' node of the workflow, while 'Visualization' plugins are used as visualization tools in the 'Visualization' node of the workflow. For dependency files, users should upload the files prepared in the previous step as follows: `requirements.txt`, `environment.yml`, and `renv.lock` files should be uploaded to 'Standard Dependency Files'; binary archives (e.g., `.whl`, `.tar.gz`) should be uploaded to 'Local Dependency Files'; and custom modules can be uploaded as folders to 'Custom Script Folder'. All dependencies required for script execution must be uploaded.

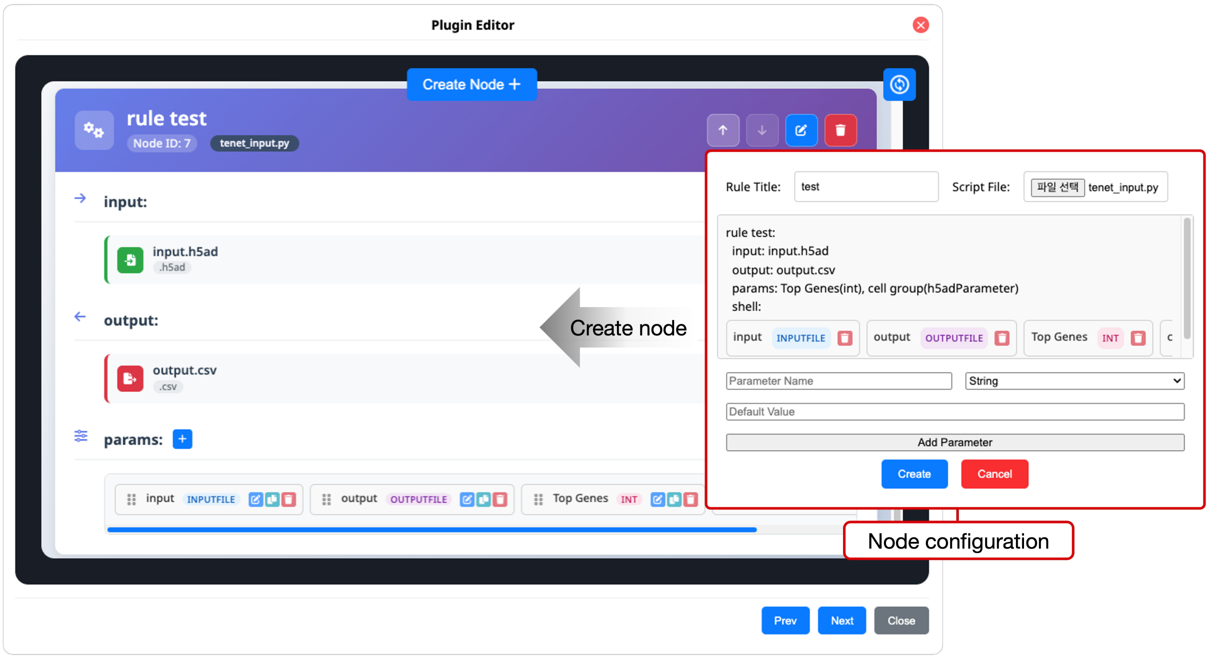

Figure N6. An example of the GUIs for plugin registration step 2.

When defining nodes through the 'Plugin editor', each node must include a unique rule name, input and output file declarations using 'Input File' and 'Output File', and parameters with specified data types, default values, and applicable constraints such as minimum/maximum ranges or file extension filters.

All file paths must be designated as either 'Input File' or 'Output File', with optional file inputs specified as 'Input File (Optional)'. For parameters, the type must be selected from 'String', 'Integer', 'Float', or 'Boolean', and a Default Value must be defined. The Default Value will be displayed as the recommended parameter setting when used in the Workflow.

When defining 'Input File', 'Output File', and 'Input File (Optional)', it is important to match filenames to enable automatic data connection between nodes. For example:

- `Node A: OutputFile = "File1.csv", Node B: InputFile = "File1.csv"`

When input/output file names are defined according to this convention, connection lines between nodes will appear in the 'Plugin editor'. This indicates that the data flow between plugin nodes is properly connected.

Plugins may also include preprocessing steps to handle H5AD files, particularly for extracting and formatting subsets of single-cell data for downstream GRN inference. The selection process is typically controlled through the following parameters:

- **cell group**: A column name in the annotation data that indicates cell types.
- **pseudotime column**: A column that represents pseudotime values for each cell.
- **clusters**: A list of clusters to include, specified as a semicolon-separated string (e.g., `"1;3;5"`).
- **UMAP lasso**: A parameter that allows users to select cells interactively using a lasso tool in UMAP space.

These parameters are utilized to generate three key output files: `expression.csv`, a gene expression matrix in `cell` × `gene` format; `trajectory.txt`, a vector of pseudotime values; and `cellSelect.txt`, a binary mask indicating selected cells (0 for unselected, 1 for selected). These files can be passed directly into GRN inference modules.

When the plugin type is set to 'Visualization', users must define an 'Output File' for all nodes to produce a single JSON format file named after the node's rule name as the script output. For example, visualization scripts implemented in Python or R must be modified to export visualization results as JSON format files using libraries such as Plotly or ggplot2. This is a required convention to ensure that nodes within the plugin function as visualization tools and render interactive results directly within the web interface.

Through this process, users can specify script files, input/output files, and parameters for all nodes within the plugin, which are then viewable in Snakefile rule format.

Step 3: Testing the plugin

Once all nodes and rules have been defined, the plugin must be reviewed for correctness and compatibility within the CellCraft environment (Figure N6). As the final step of the plugin registration process, users should navigate to the 'Validate and Upload Plugin' view to inspect all plugin metadata and rule definitions. After confirming that all settings are accurate, the plugin can be uploaded.

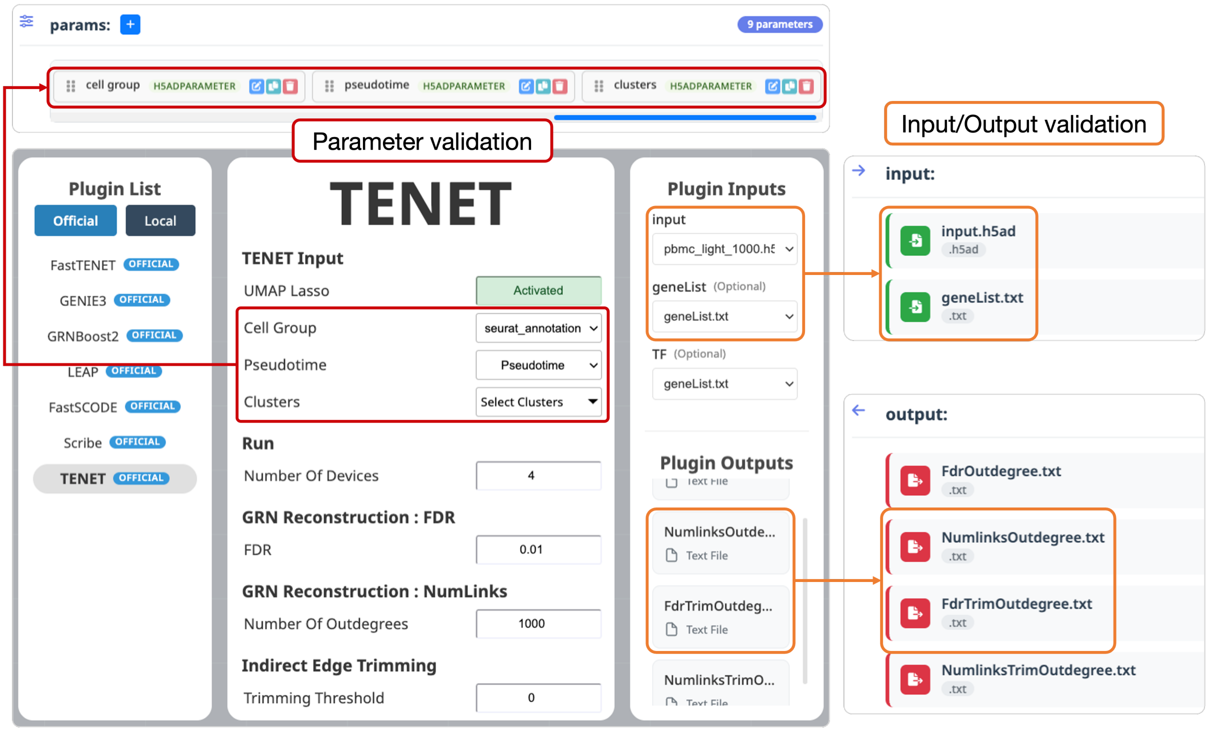

Figure N7. An example of GUIs for plugin validation.

Following a successful upload, the plugin should be tested within the Workflow editor. For 'Analysis' type plugins, users are advised to create a workflow project based on the registered plugin and inspect the included 'Algorithm' nodes. For 'Visualization' type plugins, users should set result data generated from existing 'Analysis' plugins in the 'ResultFiles' node and connect it to the 'Visualization' node for testing. At this stage, it is important to verify that the rule parameters and defined plugin inputs and outputs align correctly with the intended specifications.

After completing the testing process, users should execute tasks through the plugin and verify in the 'Task monitoring' panel that the task status shows 'SUCCESS' and that result data is generated. If errors occur during execution, it is recommended to check which stage of the plugin encountered the error and what error context was output using the 'View Logs' and 'View Progress' features. Errors may result from various causes, including improperly configured execution environments, incorrect logic in execution scripts, improper definitions of input/output files and parameters, or insufficient resources. In such cases, users are advised to consult the troubleshooting guide in the official documentation. Alternatively, users can create an issue including error logs in the official GitHub repository for resolution. To modify plugins based on troubleshooting results, users should visit the 'Plugins' page and click the edit button (gear icon) for the tested plugin, following the same process described in the Adding custom plugins section. It is crucial to delete the problematic elements (dependency files, script files, parameters, etc.) and replace them with new ones.
